# Supplementary material for: Cancer-associated TERT promoter mutations abrogate telomerase silencing
Source: eLife. 2015 Jul 21;4:e07918. doi: 10.7554/eLife.07918 (PMC4507476; doi:10.7554/eLife.07918)
Supplement: Supplementary file 1. — Summary of qRT-PCR primers used in this study. DOI: http://dx.doi.org/10.7554/eLife.07918.017 [file elife07918s001.docx]

Supplement table 1

| **qRT-PCR primers** |  |
| --- | --- |
| hTERT_qPCR_Fw | TGTCAAGGTGGATGTGACGG |
| hTERT_qPCR_Rev | GAGGAGCTCTGCTCGATGAC |
| hGAPDH1_qPCR_A | CAGTCTTCTGGGTGGCAGTGA |
| hGAPDH1_qPCR_S | CGTGGAAGGACTCATGACCA |
| OCT4_qPCR_A | CGTTGTGCATAGTCGCTGCT |
| OCT4_qPCR_S | GCTCGAGAAGGATGTGGTCC |
| TUJ1_qPCR_Fw | GGCCAAGGGTCACTACACG |
| TUJ1_qPCR_Rev | GCAGTCGCAGTTTTCACACTC |
| COL1A1_qPCR_Fw | GTCACCCACCGACCAAGAAACC |
| COL1A1_qPCR_Rev | AAGTCCAGGCTGTCCAGGGATG |
| hNanog_qPCR_A | GCAGAAGGCCTCAGCACCTA |
| hNanog_qPCR_S | AGGTTCCCAGTCGGGTTCA |
| BrachyuryT_qPCR_Fw | ACCCAGTTCATAGCGGTGAC |
| BrachyuryT_qPCR_Rev | CCATTGGGAGTACCCAGGTT |
| Nestin_qPCR_Fw | CTGCTACCCTTGAGACACCTG |
| Nestin_qPCR_Rev | GGGCTCTGATCTCTGCATCTAC |
| TR_qPCR_Fw | CCCTAACTGAGAAGGGCGTA |
| TR_qPCR_Rev | AGAATGAACGGTGGAAGGCG |

­­
